# Soy consumption and serum uric acid levels: A systematic review and meta-analysis

**DOI:** 10.3389/fnut.2022.975718

**Published:** 2022-09-02

**Authors:** Ying Duan, Qi Qi, Zihao Liu, Min Zhang, Huaqing Liu

**Affiliations:** ^1^School of Public Health, Bengbu Medical College, Bengbu, Anhui, China; ^2^School of Health Management, Bengbu Medical College, Bengbu, Anhui, China

**Keywords:** animal trials, clinical studies, meta-analysis, soy, systematic review, uric acid

## Abstract

**Background:**

Soy consumption has health benefits, but the relationship between soy and uric acid remains uncertain. This meta-analysis and systematic review evaluated the effects of soy intake on plasma uric acid.

**Methods:**

PubMed, Embase, CNKI, and the Cochrane Library were searched for studies evaluating the effects of soy, soy products, soy protein, and soy isoflavones on uric acid levels. The primary outcome was serum or plasma uric acid concentration. Study quality was evaluated by the Cochrane Collaboration and SYRCLE risk-of-bias tools.

**Results:**

A total of 17 studies were included. Qualitative analysis of three human clinical studies of acute effects revealed that soy consumption increased serum uric acid concentration; however, soy-derived products, including tofu, bean curd cake, and dried bean curd sticks, had no significant effect on serum uric acid. A meta-analysis of five long-term human studies (10 data sets) revealed that soy protein and soy isoflavones had no significant effects on uric acid levels [weighted mean difference (WMD) = –2.11; 95% confidence interval (CI): –8.78, 4.55; *p* = 0.53]. However, most epidemiological data revealed that soy intake is inversely associated with uric acid levels. Meta-analysis of nine animal trials (29 data sets) revealed that soy protein and soy isoflavones significantly reduced serum uric acid concentrations (vs. controls; MD = –38.02; 95% CI: –50.60, –25.44; *p* < 0.001).

**Conclusion:**

Soy and its products have different effects on serum uric acid. Soy products like tofu, bean curd cake, and dried bean curd sticks could be high-quality protein sources for individuals with hyperuricemia or gout. It can be beneficial to nutritionists and healthcare decision-makers reconsider their conceptions about the relationship between soy and uric acid levels according to the latest and further scientific study results.

**Systematic review registration:**

[www.crd.york.ac.uk/PROSPERO], identifier [CRD42022331855].

## Introduction

Soybeans, a member of the Fabaceae (legume) family, contain all eight essential amino acids (1, 2). In Asian countries, soy and its products have been consumed for centuries. In recent decades, the production and consumption of soy foods in Western countries have grown due to their reported health benefits (3). Some countries had approved health claims for soy protein, including the United States, Japan, South Africa, Philippines, Brazil, Malaysia, South Korea, Indonesia, and the United Kingdom (4, 5). Soy is a popular food due to its nutritional value, and it provides a wide range of health benefits.

Intake of soy protein or isoflavones is often used as an index of soy consumption because of the relevance of protein and isoflavone fractions and their variability in soy foods (6). Daily soy protein intake of up to 17 g was reported among women with plant-based dietary habits in Shanghai (7). Among adult people in Japan, the average daily intake range of soy protein, between 6 and 11 g, and isoflavone intake is 23 and 54 mg (8). The average soy, soy protein, and soy isoflavone intake of 47 Japanese prefectures from 1980 to 1985 was reported to be 66.8 g, 6.5 g, and 27.8 mg, respectively (9). In Japan, the daily median intake of daidzein and genistein (10) was reported to be 9–12 and 15–20 mg, respectively (11). Daily isoflavone intake has been estimated at 0.3–4.5 mg in European countries and approximately 1–3 mg in the United States (12–16). Moreover, sales of United States soy foods doubled in 6 years, from $2 billion in 1999 to $4.3 billion in 2005. Food manufacturers in the United States introduced more than 2,700 new foods with soy as an ingredient from 2000 to 2007, including 479 in 2006 alone (17).

Hyperuricemia, a risk factor for gout (18), results from increased uric acid production or/and impaired renal uric acid excretion. Uric acid is the final enzymatic product of purine metabolism. Ingesting foods rich in purines may increase serum and plasma uric acid concentrations (19).

The purine content in soy is approximately 137 mg/100 g (20, 21). Soy foods are generally considered to have moderate purine content, at 50–150 mg/100 g (22). High-protein foods also have high purine content. Adenine and guanine concentrations aid in controlling protein biosynthesis (23). Soy contains 35–40% protein by dry weight. Because soy proteins contain all essential amino acids, soy products have essentially equivalent protein value to that of animal sources but have less saturated fat and no cholesterol (24). In addition to its nutritional value, soy protein shows various biological functions, for example, anti-obesity effects and cholesterol-lowering, and may aid help to reduce the severity of lifestyle-related diseases (25–28). However, due to the relationship between protein and purine, soy protein is usually associated with increased serum or plasma uric acid.

Soy is a rich source of isoflavones (29–31). The primary isoflavones contained in soy are daidzein and genistein (32). Some studies (33, 34) revealed that a diet rich in isoflavones is associated with diminished risks of cancers, osteoporosis, and cardiovascular disease. The molecular structures of isoflavones are similar to that of the female hormone estrogen and result in a weak but similar action. Adamopoulos et al. discovered that exogenous estrogen reduced blood uric acid and promoted uric acid excretion in both men and women (35). Flavonoids have been reported to inhibit xanthine oxidase activity *in vitro* (36, 37). Soy isoflavones may help to reduce serum and plasma uric acid levels.

Although epidemiological studies have linked many potential benefits to soy intake (38), health professionals, and the general public in Asia broadly believe that soy foods increase the risk of gout. To date, little research has investigated the effects of soy and its related substances on serum or plasma uric acid. Moreover, findings concerning the relationship of soy and its related substances with serum and plasma uric acid levels, hyperuricemia, and gout are inconsistent. Accordingly, this review evaluates the effects of soy, soy products, soy protein, and soy isoflavones on serum and plasma uric acid levels through meta-analyses and a systematic evaluation of literature from Asian and Western countries with high soy consumption.

## Methods

### Literature search

This systematic review was carried out according to the Preferred Reporting Items for Systematic Reviews and Meta-Analyses (39). We conducted a thorough literature search of PubMed, Embase, CNKI, and the Cochrane Library to identify human and animal studies on the effects of soy consumption on serum or plasma uric acid. Our search terms included combinations of the following keywords and their variants: *uric acid*, *soy*, *isoflavones*, *soy protein*, *daidzein*, and *genistein*. No language or date restrictions were applied. The keyword search yielded a comprehensive list of titles and abstracts, which were screened for relevance against the study selection criteria mentioned in the following section. Two reviewers (YD and QQ) individually considered the full text for inclusion, and any discrepancies were resolved through consensus.

### Study selection criteria

**FIGURE 1:**
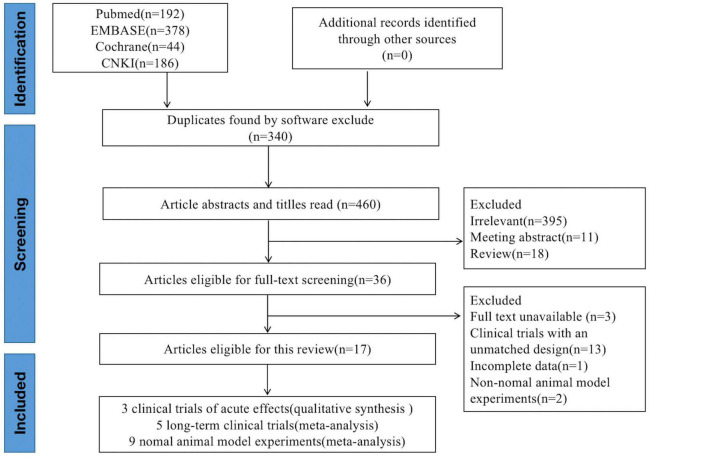
Literature selection process for the present systematic review and meta-analysis.

To meet the inclusion criteria, studies must be (a) clinical studies or animal control trials of (b) participants or animals with normal uric acid levels, (c) group serum or plasma uric acid levels must be reported as means ± standard deviations (SD; clinical trials: at baseline and the end of the trial; animal trials: at the end of the trial), and (d) data on soy, soy product, soy protein, or isoflavone intake must be reported. Meeting summaries, letters to the editor, patents, review articles, unpublished articles, articles with incomplete data, and articles without available full texts were excluded. A flowchart of the screening and selection process is depicted in Figure 1.

### Risk of bias

The risk of bias was evaluated using the Cochrane Collaboration tool (40) for randomized clinical trials and the Systematic Review Center for Laboratory animal Experimentation (SYRCLE’s) Risk of Bias (RoB) tool (41) for animal studies. The Cochrane Collaboration tool assesses (a) outcome reporting, (b) blinding, (c) allocation concealment, (d) outcome data completeness, (e) randomization, (f) and other sources of bias. The SYRCLE risk-of-bias tool is a version of the Cochran tool modified for animal intervention studies. It assesses (a) baseline characteristics, (b) sequence generation, (c) allocation concealments (d) housing randomness, (e) blinding (performance bias), (f) blinding (detection bias), (g) outcome assessment randomness, (h) outcome data completeness, (i) outcome reporting, and (j) other sources of bias.

The two reviewers independently evaluated the risk of bias for each eligible study and disagreements on scores were resolved through consensus.

### Data extraction

The two reviewers independently identified the titles and abstracts that potentially met the inclusion criteria. Then, the full-text articles were read for complete assessment and selection. Each reviewer performed these steps independently, and disagreements on inclusion were resolved through consensus. Data for each included article were recorded, including the year of publication; name of the first author; study period; group size; age; sex; and exposure to soy, soy products, soy protein, or soy isoflavones.

### Statistical analysis

RevMan v5.4 (Cochran Tech, London, United Kingdom) was used for meta-analysis and the creation of forest plots. The units of uric acid concentration were converted to μmol/L.

For animal studies, random effects models were used throughout due to the possible heterogeneity from sources, such as differences in the time at which the endpoints, were evaluated. In addition, the *I*^2^ statistic was used to assess heterogeneity within subgroups. *I*^2^ values > 25% and > 75% represented moderate and high heterogeneity, respectively. Mean difference (MD) and 95% confidence intervals (CIs) were used to determine the effect.

For clinical studies, heterogeneity between trials was detected with the chi-square and *I*^2^ tests. In the situation of significant heterogeneity (*I*^2^ > 50% and *p* < 0.1), a random-effects model was performed for analysis; otherwise, a fixed effects model was performed. The weighted mean difference (WMD) and 95% CI were used to determine the effects. If the mean or SD was unavailable, it was calculated from other mean values and standard error by using the following formulas (assuming a correlation coefficient *R* = 0.5 (42):


(1) mean=mean2-mean1⁢S⁢D=(n1-1)⁢S⁢D12+(n2-1)⁢S⁢D22n1+n2-2

where mean_1_ is the preintervention mean; mean_2_ is the postintervention mean; *n*_1_ is the preintervention sample size; *n*_2_ is the postintervention sample size; *SD*_1_ is the preintervention SD; and *SD*_2_ is the postintervention SD. Studies with *p* < 0.05 were considered statistically significant, and two-sided 95% CIs were employed. For studies that could not be analyzed through meta-analysis, descriptive analysis and evaluation (i.e., qualitative analysis) were performed.

## Results

### Human studies

Eight (43–50) human clinical studies met the inclusion criteria. These were categorized into those assessing acute or long-term effects based on the study period. Specifically, three (43–45) assessed acute effects and five (46–50) assessed long-term effects. Table 1 displays the details of these studies.

**TABLE 1:** Characteristics of human clinical studies.

Study	Nation	Subject	Age (years)	Sample	Intervention	Acute or long-term effects	Research type
							
Zhang et al. (43)	China	Healthy Adult men	Unknown	60	Whole soy, soy milk, soy power, bean curd cake and dried bean curd stick	Acute effects	Randomized controlled trial
Yamakita et al. (44)	Japan	8 healthy male and 10 male patients with gout	30–50	18	Tofu (bean curd)	acute effects	before-after study in the same patient
Garret et al. (45)	Canada	Healthy volunteers	24.8 ± 2.1	10	Soy Protein	Acute effects	Randomized controlled crossover trial
Berg et al. (46)	Germany	Healthy sports students	23.6 ± 1.9	30	Soy Protein	Long-term effects	Randomized controlled trial
Chen et al. (47)	the United States	Non-smoking healthy man	20 ± 1.94	30	Soy isoflacone	Long-term effects	Randomized controlled trial
Liu et al. (48)	China	Postmenopausal Chinese women with prediabetes or early untreated diabetes	48–70	120	Soy isoflacone	Long-term effects	Randomized controlled trial
Qin et al. (49)	China	Patients with hypercholesterolemia	40–65	120	Daidzein	Long-term effects	Randomized controlled trial
Ye et al. (50)	China	Women with impaired glucose regulation	30–70	165	Daidzein	Long-term effects	Randomized controlled trial

#### Acute effects of soy, soy products, and soy protein on plasma uric acid concentrations

Of the three studies of acute effects, two (43, 44) focused on the effects of soy or soy products on uric acid and one (45) focused on the effects of soy protein. Due to differences in research design (randomized controlled trial, before-after study in the same patient, and randomized controlled crossover trial, respectively), reliable meta-analysis was impossible; therefore, qualitative analysis was performed.

The first study (43) considered was a randomized controlled study conducted by Zhang et al. to detect the serum uric acid concentrations of 60 healthy men after they ingested whole soy, one of four soy products (soy milk, soy powder, bean curd cake, or dried bean curd sticks), or water. The results revealed no significant changes from baseline in serum uric acid concentration after the ingestion of dried bean curd sticks, bean curd cake, or water (*p* > 0.05). After ingestion of whole soy, serum uric acid concentration significantly increased in the short term, at 21.4 μmol/L after 60 min and 16.3 μmol/L after 120 min; although uric acid remained high through 180 min, this difference was not statistically significant from baseline. The same significant results occurred after the ingestion of soy powder. For soy milk, serum uric acid concentration had increased and was found to be 38.1, 34.4, and 24.1 μmol/L at 60, 120, and 180 min after ingestion, respectively. However, bean curd cake induced a significant decrease in serum uric acid at 60 min in participants with high serum uric acid concentrations.

Another study (44) examined the effect of tofu ingestion on the uric acid metabolism of 8 healthy participants and 10 participants with gout, all aged 30–50 years. Plasma levels (mg/dL) at 0, 60, 120, and 180 min after the ingestion of 4 g/kg of tofu by healthy participants and participants with gout were 5.56, 5.59, 5.83, 5.73, and 8.10, 8.21, 8.27, and 8.12 mg/dL, respectively. Plasma uric acid levels in healthy patients increased statistically after 120 min and 180 min compared with that at baseline, whereas the participants with gout exhibited no significant differences. The plasma uric acid concentrations of the healthy participants and those with gout also did not differ significantly. These results suggest that tofu is a preferable source of protein.

Garrel et al. (45) examined the acute effects of ingesting 80 g of casein, lactalbumin, or soy isolate protein on serum uric acid concentrations. The cohort comprised 10 healthy participants aged 22–27 years. Serum uric acid levels at 0, 60, 120, and 180 min after soy isolate protein ingestion were 283, 307, 319, and 314 μmol/L (*p* < 0.01), respectively.

The results of these three studies revealed that ingesting soy or soy protein rapidly increases serum uric acid. However, tofu, bean curd cake, and driven bean curd sticks had no significant effect on uric acid concentration.

#### Long-term effects of soy protein and isoflavones on plasma uric acid concentrations

Five studies of long-term effects met the inclusion criteria. One (46) focused on soy protein and four (47–50) focused on soy isoflavones; of these four, two (47, 48) studied unspecified soy isoflavones, one (49) studied daidzein, and one (50) studied genistein. Some studies employed multiple doses or intervention expectations, and we treated these as individual trials. Thus, this category comprised 10 trials. Table 2 presents the data extracted from each study.

**TABLE 2:** Characteristics of long-term human clinical studies.

Study	Subject	Age (years)	Gender (M:F)	Soy group				Control group				Study period (weeks)
				Sample size	Age (years)	Gender (M:F)	Intervention	Sample size	Age (years)	Gender (M:F)	Intervention	
Berg et al. (46)	Healthy Sports students	23.6 ± 1.9	20/10	14 (Baseline 15)	23.3 ± 1.6	10/5	Soy protein 53.3 g/d	14 (Baseline 15)	24.0 ± 2.1	10/5	blank	6
Chen et al. (47)	Non-smoking Healthy man	20 ± 1.94	30/0	15	20.7 ± 2.4	15/0	Soy isoflavones 150 mg/d	15	20.7 ± 1.5	15/0	placebo	4
Liu et al. (48)	Postmenopausal Chinese women with prediabetes or early untreated diabetes	48–70	0/120	60	50.6 ± 3.4	0/60	Soy isoflavones 100 mg/d	60	55.9 ± 3.8	0/60	milk protein	12
	Postmenopausal Chinese women with prediabetes or early untreated diabetes	46–70	0/120	60	50.6 ± 3.4	0/60	Soy isoflavones 100 mg/d	60	55.9 ± 3.8	0/60	milk protein	24
Qin et al. (49)	Patients with hypercholesterolemia	40–65	55/62	58 (Baseline 60)	54.5 ± 6.6	28/30	Daidzein 40 mg/d	59 (Baseline 60)	52.9 ± 6.0	27/32	placebo	12
	Patients with hypercholesterolemia	40–65	53/56	60	53.4 ± 6.4	36/24	Daidzein 80 mg/d	59 (Baseline 60)	52.9 ± 6.0	27/32	placebo	24
Ye et al. (50)	Women with impaired glucose regulation	30–70	0/104	54 (Baseline 55)	53.4 ± 6.4	0/54	Daidzein 50 mg/d	50 (Baseline 54)	56.3 ± 11.1	0/50	placebo	12
	Women with impaired glucose regulation	30–70	0/97	50 (Baseline 55)	53.4 ± 6.4	0/50	Daidzein 50 mg/d	47 (Baseline 54)	56.3 ± 11.1	0/47	placebo	24
	Women with impaired glucose regulation	30–70	0/104	54 (Baseline 56)	53.4 ± 6.4	0/54	Genistein 50 mg/d	50 (Baseline 54)	56.3 ± 11.1	0/50	placebo	12
	Women with impaired glucose regulation	30–70	0/101	54 (Baseline 56)	53.4 ± 6.4	0/54	Genistein 50 mg/d	47 (Baseline 54)	56.3 ± 11.1	0/47	placebo	24

### Effect assessment

**FIGURE 2:**
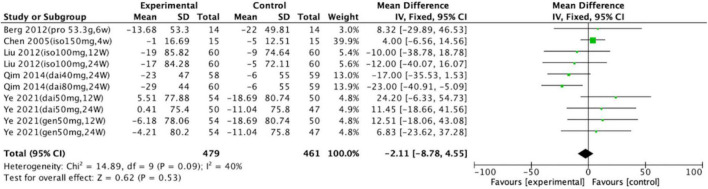
Forest plot of mean change in uric acid concentration after intervention with soy protein and soy isoflavones (SD, standard deviation; CI, confidence interval; IV, inverse variance; μmol/L).

Of the 10 trials, only one revealed significant differences between the control and intervention groups. In the study by Qim et al., uric acid concentration decreased by 23 μmol/L after intervention with 80 mg of daidzein for 24 weeks (intervention group, *n* = 60; control group, *n* = 59, *p* = 0.001; 95% CI: –40.91, –5.09). The 10 trials (with a total of 940 participants) that compared a soy protein or soy isoflavone group with a blank or placebo control group were meta-analyzed. The heterogeneity results were *x*^2^ = 14.89, *p* = 0.09, and *I*^2^ = 40%. A fixed-effects model analysis indicated no statistically significant differences in the long-term effects of soy protein and soy isoflavones on the uric acid levels of intervention groups compared with those of the blank and placebo groups (*p* = 0.53; Figure 2).

**FIGURE 3:**
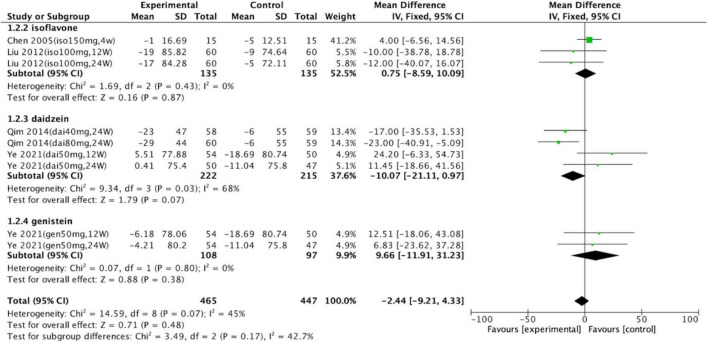
Forest plot of mean change in uric acid concentration after intervention with soy isoflavones. Subgroup analyses evaluated the effects of daidzein, genistein, and unspecified soy isoflavones (SD, standard deviation; CI, confidence interval; IV, inverse variance; μmol/L).

Subgroup analyses were conducted to assess the effects of isoflavones on serum uric acid levels (Figure 3). Groups consuming daidzein (intervention group, *n* = 222; control group, *n* = 215; *p* = 0.07; WMD = –10.07; 95% CI: –21.11, 0.97), genistein (intervention group, *n* = 108; control group, *n* = 97; *p* = 0.38; WMD = 9.66; 95% CI: –11.91, 31.23), or soy isoflavones (intervention group, *n* = 135; control group, *n* = 135; *p* = 0.87; WMD = –0.75; 95% CI: –8.59, 10.09) did not significantly differ from the control groups. Total meta-analysis revealed that soy isoflavones had no effect on uric acid levels (intervention group, *n* = 465; control group, *n* = 447; *p* = 0.48; WMD = –2.44; 95% CI: –9.21, 4.33).

### Risk of bias of studies of long-term effects

**FIGURE 4:**
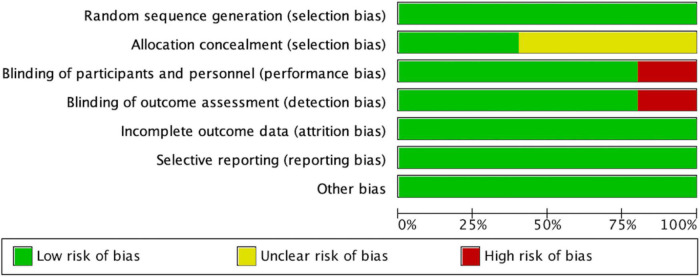
Risk of bias graph, with risk of each type of bias presented as a percentage among included studies).

**FIGURE 5:**
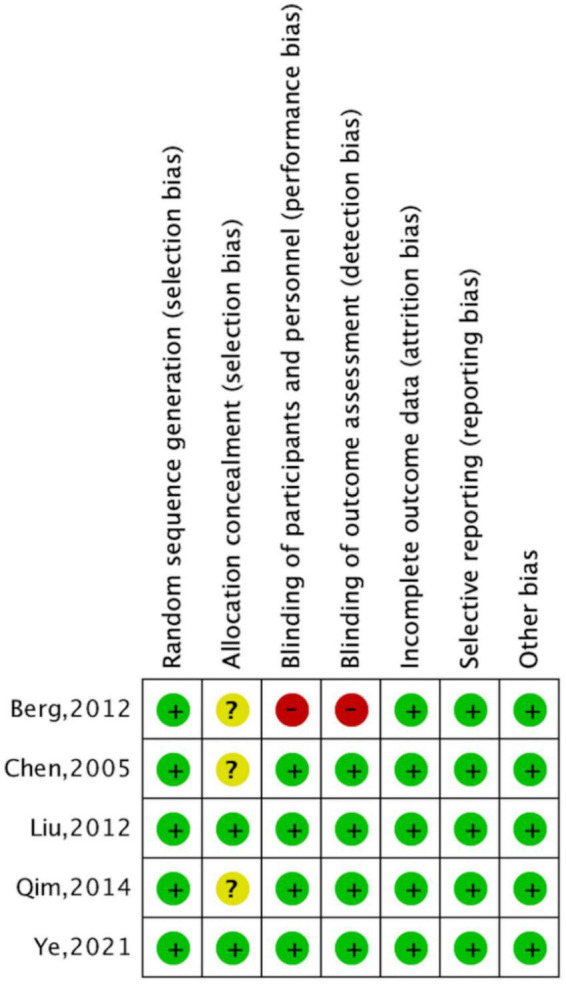
Risk of bias summary, with risk of each type of bias in included studies presented separately. Note: +, ?, – indicate high, uncertain, and low bias, respectively.

According to the Cochrane Collaboration risk-of-bias assessment, the five studies included failed to achieve all seven benchmarks. The overall risks for each type of bias are presented in Figure 4, and the risks of each bias for each included study are presented in Figure 5. One study (20%) exhibited high risks of performance bias and detection bias. Three studies (60%) demonstrated unclear risks of selection bias (allocation concealment).

#### Epidemiology of soy consumption and serum uric acid levels

To better evaluate the relationship between soy consumption and serum uric acid in real life, we reviewed the epidemiological data. An earlier study conducted by Pan et al. interviewed 59 non-vegetarian medical students and 55 vegetarian Taiwanese Buddhists (51). In which, non-vegetarians consumed one serving of soy food 1 day, while vegetarians consumed 3.5 servings. The results revealed that the plasma uric acid level of vegetarians was significantly lower than that of non-vegetarians (226 ± 59 mmol/L and 258 ± 54 mmol/L, respectively) in women, but, no such difference in men. Villegas et al. conducted a cross-sectional study among 3,978 men in Shanghai (52). The results revealed that intake of soy products (tofu, fried tofu, vegetarian chicken, and tofu cake) was associated with a decreased risk of hyperuricemia. Another cross-sectional study by Liu et al. was conducted among 2,939 adults aged 50–75 in Guangzhou (53). The participants were divided into quartiles according to their intake amount of soy protein. Soy protein from soy, soy drink, and other soy products was negatively correlated with the risk of hyperuricemia. Tang et al. divided 120 volunteers aged 54–56 years into a control group and a genistein-rich group (54) and observed a significant difference in serum uric acid levels between the two groups (5.34 ± 1.13 mg/dL in the control group and 4.75 ± 1.21 mg/dL in genistein-rich group). A large cohort study (55) collected data on the eating habits of 63,257 Singaporean Chinese aged 45–74 years, and found that soy was associated with a decreased risk of gout (hazard ratio = 0.86, 95% CI: 0.75–0.98). However, inconsistent with these results, a previous study by Yu et al. (56) assessed the intake of soy beverages among 987 men and 1,189 women and revealed that the intake of soy beverages was not associated with the risk of hyperuricemia. Liu et al. (57) also conducted a cross-sectional study about soy isoflavones supplementation among 183 Chinese adults. Their study observed no significant difference in serum uric acid levels between low isoflavones groups with the consumption of isoflavones of 4.6 mg/d and high isoflavones groups with 23.6 mg/d (274.67 ± 99.00 μmol/L and 269.01 ± 84.88 μmol/L, respectively).

### Animal studies

#### Characteristics of included studies

Nine studies (58–66) met the inclusion criteria. Two (58, 59) studied the effects of soy protein and the remaining seven (60–66) studied the effects of soy isoflavones on uric acid. Among the seven studies on isoflavones, three (61, 64, 65) focused on daidzein, three (60, 62, 63) on genistein, and one (66) on unspecified soy isoflavones. As with long-term clinical studies, some studies employed different doses and intervention periods, and we divided the corresponding research into individual trials. Thus, this category comprised 29 trials. Table 3 displays the details of these trials.

**TABLE 3:** Characteristics of animal trials.

Study	Animal species	Age (week)	Soy active ingredients	Control	Soy active dosage	Study period (days)	Sample
Bhathena et al., (58)	Rat	8–10	Soy Protein	Casein	< 1 mg/kg/d	180	16
Cui (59)	Rat	Unknown	Soy Protein	Casein	11% (percentage of mass fraction in total mass fraction of feed)	28	16
	Rat	Unknown	Soy Protein	Casein	22% (percentage of mass fraction in total mass fraction of feed)	28	16
	Rat	Unknown	Soy Protein	Casein	44% (percentage of mass fraction in total mass fraction of feed)	28	16
Palanisamy et al. (60)	Rat	Adult	Genistein	Starch	1 mg/kg/d	60	12
Karale and Kamath (61)	Rat	Adult	Daidzein	Normal Saline	40 mg/kg/d	10	12
Huang et al. (62)	Mice	Unknown	Genistein	Distilled Water	4.5 mg/kg/d	7	20
	Mice	Unknown	Genistein	Distilled Water	9 mg/kg/d	7	20
	Mice	Unknown	Genistein	Distilled Water	18 mg/kg/d	7	20
Huang et al. (63)	Mice	Unknown	Genistein	Distilled Water	4.5 mg/kg/d	7	20
	Mice	Unknown	Genistein	Distilled Water	9 mg/kg/d	7	20
	Mice	Unknown	Genistein	Distilled Water	18 mg/kg/d	7	20
Zhao et al. (64)	Laying hens	44	Daidzein	Blank	5 mg/kg/d	28	26
	Laying hens	44	Daidzein	Blank	10 mg/kg/d	28	26
	Laying hens	44	Daidzein	Blank	15 mg/kg/d	28	26
	Laying hens	48	Daidzein	Blank	5 mg/kg/d	28	26
	Laying hens	48	Daidzein	Blank	10 mg/kg/d	28	26
	Laying hens	48	Daidzein	Blank	15 mg/kg/d	28	26
	Laying hens	52	Daidzein	Blank	5 mg/kg/d	28	26
	Laying hens	52	Daidzein	Blank	10 mg/kg/d	28	26
	Laying hens	52	Daidzein	Blank	15 mg/kg/d	28	26
Meng et al. (65)	Laying hens	55	Daidzein	Blank	3 mg/kg/d	14	16
	Laying hens	55	Daidzein	Blank	15 mg/kg/d	14	16
Wang et al. (66)	Broiler	3	Soy isoflavones	Blank	3 mg/kg/d	10	10
	Broiler	3	Soy isoflavones	Blank	6 mg/kg/d	10	10
	Broiler	3	Soy isoflavones	Blank	9 mg/kg/d	10	10
	Broiler	3	Soy isoflavones	Blank	3 mg/kg/d	21	10
	Broiler	3	Soy isoflavones	Blank	6 mg/kg/d	21	10
	Broiler	3	Soy isoflavones	Blank	9 mg/kg/d	21	10

#### Effect assessment

The effects of soy protein and soy isoflavones on serum and plasma uric acid levels were reported for the 29 data sets of the nine studies (intervention group, *n* = 267; control group, *n* = 67). The meta-analysis indicated that, compared with the levels in the controls, soy protein and isoflavones were resulted in lower serum and plasma uric acid levels (*p* < 0.001; MD = –38.02; 95% CI: –50.60, –25.44).

**FIGURE 6:**
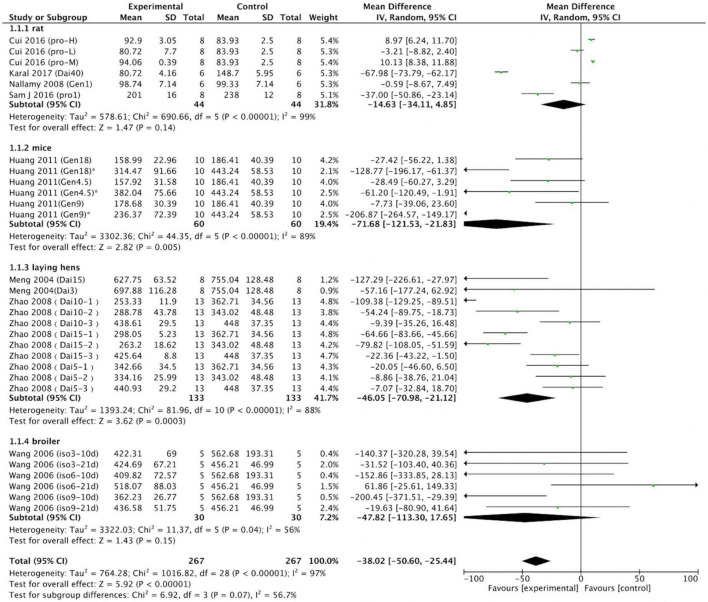
Forest plot of the effects of soy protein and soy isoflavones on serum and plasma uric acid concentration (grouped by animal species; SD, standard deviation; CI, confidence interval; IV, inverse variance; μmol/L).

We performed further subgroup analysis. First, a meta-analysis was carried out on rats, mice, laying hens, and broiler chickens. Six rat trials (control group, *n* = 44; intervention group, *n* = 44) were analyzed. Four trials revealed differences between the control and intervention groups. Two intervention groups exhibited significantly increased serum or plasma uric acid levels, whereas the other two intervention groups exhibited reduced serum and plasma uric acid concentrations. A meta-analysis of six rat trials revealed that the effect of the intervention group on reducing uric acid was non-significant (*p* = 0.14). Six mouse trials (*n* = 60 in control group; *n* = 60 in intervention group) were analyzed. In five of the six trials, mice receiving soy protein or isoflavones exhibited reduced uric acid levels; in the remaining trial, no differences were observed between the intervention and control animals. Overall, soy protein or soy isoflavones reduced serum and plasma uric acid (MD = –71.68; 95% CI: –121.53, –21.83; *p* = 0.005) in mice; this result differs from that of the rat trials. Eleven laying hen trials were included in the meta-analysis. In five of these trials, soy protein or soy isoflavones reduced uric acid levels, whereas none was observed in the other six trials. Of 133 intervention and 133 control laying hens, soy protein or soy isoflavones reduced uric acid levels by a mean of 46.05 μmol/L (95% CI: –70.98, –21.12; *p* < 0.001). Six broiler chicken trials (*n* = 30 in the control group; *n* = 30 in the intervention group) analyzed the effect of soy protein or soy isoflavones on uric acid levels. In three of these trials, broilers receiving soy protein or soy isoflavones exhibited reduced uric acid levels, whereas no differences were observed between the intervention and control animals in the other three trials. Meta-analysis of these broiler trials revealed that soy protein or soy isoflavones had no effect on serum or plasma uric acid levels (*p* = 0.15; Figure 6).

**FIGURE 7:**
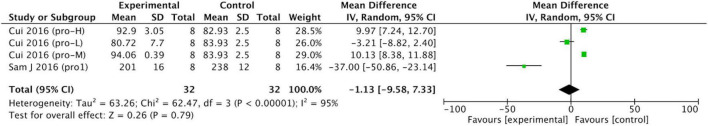
Forest plot of the effects of soy protein (SD, standard deviation; CI, confidence interval; IV, inverse variance; μmol/L).

We separated the data into those on soy protein and those on soy isoflavones for meta-analysis. Soy protein (*p* = 0.79; MD = –1.13; 95% CI: –9.58, 7.33) had no effect on uric acid concentration (Figure 7).

**FIGURE 8:**
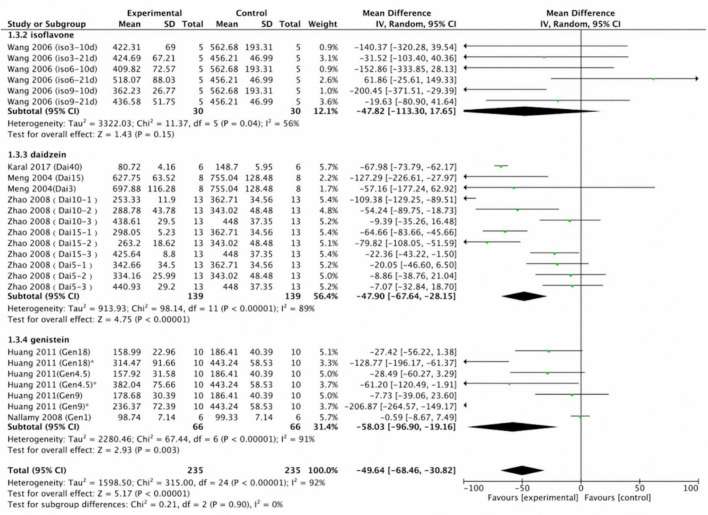
Forest plot of the effects of soy on serum and plasma uric acid concentration. Subgroup analyses evaluated the effects of daidzein, soy protein, genistein, and unspecified soy isoflavones (SD, standard deviation; CI, confidence interval; IV, inverse variance; μmol/L).

Subgroup analyses were performed to evaluate the effects of soy isoflavones on serum and plasma uric acid levels. Unspecified soy isoflavones (n = 30 in control group; *n* = 30 in intervention group; *p* = 0.15; MD = –47.82; 95% CI: –113.30, 17.65) did not significantly reduce serum or plasma uric acid concentrations. However, daidzein (*n* = 139 in control group; *n* = 139 in intervention group; *p* < 0.001; MD = –47.90; 95% CI: –67.64, –28.15) and genistein (*n* = 66 in control group; *n* = 66 in intervention group; *p* = 0.003; MD = –58.03; 95% CI: –96.90, –19.16) significantly reduced serum or plasma uric acid concentrations (Figure 8).

#### Risk of bias in animal studies

Eight studies (89%) mentioned random group allocation, but no studies described the randomization process. Five studies (56%) provided research animals’ baseline characteristics. None mentioned allocation concealment. Eight studies described random housing. No study described blinding (performance bias) or random outcome assessment. All studies achieved and reported complete outcome data. No other sources of bias affected any of the studies. The methodological quality of each study is displayed in Table 4.

**TABLE 4:** Risk of bias of included animal studies.

Study	(a)^a^	(b)^a^	(c)^a^	(d)^a^	(e)^a^	(f)^a^	(g)^a^	(h)^a^	(i)^a^	(j)^a^
Bhathena et al. (58)	U^k^	Y^l^	U	Y	U	U	Y	Y	Y	Y
Cui (59)	U	U	U	Y	U	U	Y	Y	Y	Y
Palanisamy et al. (60)	N^m^	Y	U	Y	U	U	Y	Y	Y	Y
Karale and Kamath (61)	U	Y	U	Y	U	U	Y	Y	Y	Y
Huang et al. (62)	U	Y	U	Y	U	U	Y	Y	Y	Y
Huang et al. (63)	U	U	U	U	U	U	Y	Y	Y	Y
Zhao et al. (64)	U	U	U	Y	U	U	Y	Y	Y	Y
Meng et al. (65)	U	U	U	Y	U	U	Y	Y	Y	Y
Wang et al. (66)	U	Y	U	Y	U	U	Y	Y	Y	Y

^a^Sequence generation; ^b^Baseline characteristics; ^c^Allocation concealment; ^d^Random housing; ^e^Blinding (performance bias); ^f^Random outcome assessment; ^g^Blinding (detection bias); ^h^Incomplete outcome data; ^i^Selective outcome reporting; ^j^Other sources of bias; ^K^Unclear, indicates an unclear risk of bias; ^L^Yes, indicates low risk of bias; ^M^No, indicates high risk of bias.

## Discussion

The relationship between soy and health has gradually become a topic of concern. The relationship between soy and uric acid levels is controversial. A common belief is that people with high serum or plasma uric acid levels should avoid eating soy due to the high purine content of soy protein, which increases the production of uric acid. However, the expected increase caused by daily intake in individuals of Asian descent is almost clinically irrelevant (67). The British Society for Rheumatology also pointed out that patients with gout should be encouraged to add vegetable- and soy-derived protein to their diets (68).

In this study, we systematically reviewed the effects of soy, soy products, soy protein, and soy isoflavones on serum and plasma uric acid levels in clinical and animal studies. Soy consumption had no significant effect on human uric acid concentration. Studies of the acute effects of soy intake on uric acid demonstrated that whole soy and soy protein increased uric acid levels in the short term. However, soy products such as tofu, tofu cake, and dried tofu had no effect. Zhang et al. (43) found that whole soy intake increased serum uric acid concentration by 6.8% after 60 min. Results from the study by Garrel et al. (45) showed that soy protein intake increased serum uric acid concentration by 12% after 120 min. This difference in serum uric acid concentration may be related to the amount of protein, with the intervention of 40 g soy protein in Zhang’s study and 80 g soy protein isolate in Garrel’s study. Consistent with the study by Garrel et al., another study (69), which was excluded from the final meta-analysis due to no report of serum uric acid concentration, observed that intake of 80 g soy protein increased serum uric acid concentration by 10% after 120 min. It is noteworthy that many studies adopted large amounts of protein (40–80 g) as intervention, which is equivalent to the daily amount of protein recommended by the Dietary Guidelines for Chinese residents, exceeding the amount typically consumed in a meal, and let alone from one protein source. In addition, Garrel’s study also examined the acute effects of casein and lactalbumin on uric acid levels. In contrast to the results of soy protein, the serum uric acid concentration of participants decreased significantly at 3 h after ingestion of casein and lactalbumin. Long-term clinical studies mainly presented evidence of the effects of soy protein and soy isoflavones on uric acid; soy had no effect on uric acid levels.

A cross-over study designed by Serrano et al. (70) investigated the effects of soy beverages on glucose homeostasis and monitored changes in serum uric acid levels. Their results revealed a significant increase in uric acid levels at 60 min after consuming soy beverage, as well as an increase in insulin levels. However, another 3-month open randomized controlled trial designed by Zhang et al. (71) revealed that soy products and fruits may help reduce the risk of asymptomatic hyperuricemia by reducing insulin resistance. Enhanced insulin sensitivity can promote the resecretion of uric acid in renal tubular epithelial cells and increase the excretion of uric acid (72). Consistent with the result, many epidemiological studies also revealed that soy consumption is inversely associated with uric acid levels.

The included animal studies evaluated the effects of soy isoflavones and soy protein on uric acid levels. For normal rats and broilers, no differences were found in uric acid concentrations between the soy intervention group and controls. For mice and laying hens, the effects of soy intervention on uric acid levels were significant, and soy isoflavones reduced uric acid concentrations. In subgroup analysis according to the type of soy, soy protein and soy isoflavones had no effect on uric acid concentration. However, daidzein and genistein, which are the main components of soy isoflavones, both significantly reduced uric acid concentrations in animal studies.

Purine in the form of purine nucleotides participates in human energy metabolism and is a crucial component of genetic material (73). Guanine and adenine are two bases of DNA and RNA (74). Protein formation depends on DNA and RNA and thus on two purines. Adenine, especially adenosine triphosphate (ATP), provides energy and can be transformed into hypoxanthine, and guanine and hypoxanthine directly synthesize xanthine. Xanthine oxidase catalyzes the oxidation of hypoxanthine and xanthine to form uric acid (75). This may explain why soy protein causes significant short-term increases in serum and plasma uric acid concentrations but no significant long-term effects.

Traditional soy products are roughly processed from soybeans as the primary raw material. The purine content of the edible portion of the soybean is affected by the water content added during processing—the lower the water content, the higher the purine content (76). Our study found that uric acid concentration increased rapidly after the intake of whole soy, whereas the intake of soy-derived products had no effect. This difference may be due to the change in purine content during soy processing.

Traditional soy foods contain approximately 3.5 mg of isoflavones per gram of protein (8). Of soy isoflavones, genistein, daidzein, and glycitein make up about 50, 40, and 10%, respectively, (10). Isoflavones have a molecular structure similar to that of estrogen, allowing them to bind to both estrogen receptors (77, 78), and to exert estrogen-like effects under certain conditions. Some studies have investigated the relationship between estrogen and uric acid. Studies have demonstrated that endogenous estrogen may reduce uric acid by inhibiting the production of uric acid reabsorption protein and promoting that of uric acid secretory protein (79). Budhiraja et al. observed the inhibition of xanthine oxidoreductase systems by estradiol in a rat model of hypoxia (80). Although the overall mechanism by which estrogen affects uric acid metabolism remains unclear, numerous studies have demonstrated that an increase in estrogen reduces uric acid levels. Soy isoflavones are commonly referred to as phytoestrogens. Circulating isoflavone levels were three orders of magnitude higher than estrogen levels after ingestion of approximately two servings of soy foods (81). Therefore, soy isoflavones may inhibit uric acid through an estrogen-like effect, and the results of our meta-analysis support this idea.

Soy is rich in protein and isoflavones. One serving of soy food provides approximately 8 g of protein and 25 mg of isoflavones. The effects of soy and its products on uric acid may be the combined effects of isoflavones and soy protein. Isoflavones and soy protein can interact. Genistein and daidzein are the two main components of aglycone-type isoflavones (82). As natural small molecules and active substances, aglycone-type isoflavones can interact with and change the structure and function of soy protein (83). The formation of soy protein and soy isoflavone complexes affects the structure and nutritional function of the protein and increases the antioxidant capacity and bioavailability of isoflavones (84, 85). However, soy protein and isoflavones have different specific effects on uric acid metabolism. Protein rich in purine may increase uric acid concentration, whereas isoflavones may reduce uric acid concentration.

## Perspectives and conclusion

Human clinical studies on the effects of soy consumption on uric acid have mostly focused on individuals with normal uric acid levels. However, the effects of a substance on serum or plasma uric acid levels are often influenced by individual differences. Statins significantly reduce serum and plasma uric acid levels in individuals with coronary heart disease complicated with dyslipidemia, and the decrease of uric acid in individuals with hyperuricemia is greater than that in patients with normal uric acid (86). Some studies have also revealed that, compared with normal rats, hyperuricemic rats exhibit significantly less and slower absorption of hesperidin (87). Therefore, it is prudent that our findings about the relationship between soy consumption and uric acid level were extended to patients with hyperuricemia or gout. Additional research is required to assess the association between soy and serum or plasma uric acid with high uric acid levels, for example, in animal models of hyperuricemia or gout. In addition, animal trials have focused on soy isoflavones, daidzein, and genistein but rarely on soy or soy protein. Accordingly, future research on soy and soy protein should be emphasized, especially in animal models of hyperuricemia and gout.

Our findings indicate that the evidence that soy increases serum or plasma uric acid concentrations remains insufficient; moreover, the relationship differs with soy products. Although soy and its active substances are believed to increase serum and plasma uric acid levels and lead to hyperuricemia or gout in Asia, the latest American College of Rheumatology (2020) and European League Against Rheumatism (2016) guidelines do not specify a maximal soy intake (88, 89). Soy products like tofu, bean curd cake, and dried bean curd sticks may be high-quality protein sources for individuals with hyperuricemia or gout. It can be beneficial to nutritionists and healthcare decision-makers reconsider their conceptions about the relationship between soy and uric acid levels according to the latest and further scientific study results.

## Data availability statement

The original contributions presented in this study are included in the article/supplementary material, further inquiries can be directed to the corresponding author/s.

## Author contributions

YD, MZ, and HL contributed to the conception and design of the study. YD, QQ, and ZL organized the data, assessed all studies for eligibility, and evaluated the risk of bias in all included studies. YD drafted the manuscript. QQ and ZL performed the statistical analyses. MZ and HL reviewed and edited the manuscript. All authors approved the final version of the manuscript.
